# Morphological changes in the spiracles of *Anopheles gambiae s.l (*Diptera) as a response to the dry season conditions in Burkina Faso (West Africa)

**DOI:** 10.1186/s13071-015-1289-0

**Published:** 2016-01-07

**Authors:** Wadaka Mamai, Karine Mouline, Jean-Philippe Parvy, Jo Le Lannic, Kounbobr Roch Dabiré, Georges Anicet Ouédraogo, David Renault, Frederic Simard

**Affiliations:** Institut de Recherche en Sciences de la Santé (IRSS), Direction Régionale de l’Ouest (DRO), 399 Avenue de la Liberté, 01 BP 545 Bobo-Dioulasso, Burkina Faso; MIVEGEC, UMR IRD 224-CNRS 5290-Université de Montpellier, Institut de Recherche pour le Développement, 911 Avenue Agropolis, BP 64501, 34394 Montpellier, cedex 5 France; Université Polytechnique de Bobo-Dioulasso (UPB), Bobo-Dioulasso, Burkina Faso; Université Pierre et Marie Curie, 75005 Paris, France; CGM, UPR 3404, CNRS, 91190 Gif-sur-Yvette, France; Université de Rennes 1, UMR CNRS 6553 ECOBIO, Campus de Beaulieu, 263 Avenue du Gal Leclerc, CS 74205 35042 Rennes, Cedex France; Institut de Recherche pour le Développement, Antenne de Bobo Dioulasso, BP 171 Bobo Dioulasso 01, Burkina Faso

**Keywords:** Spiracle, Morphology, SEM, Desiccation, *Anopheles gambiae*, Burkina Faso

## Abstract

**Background:**

Survival to dry season conditions of sub-Saharan savannahs is a major challenge for insects inhabiting such environments, especially regarding the desiccation threat they are exposed to. While extensive literature about insect seasonality has revealed morphologic, metabolic and physiological changes in many species, only a few studies have explored the responses following exposure to the stressful dry season conditions in major malaria vectors. Here, we explored morphological changes triggered by exposure to dry season conditions in *An. gambiae s.l.* mosquitoes by comparing females reared in climatic chambers reflecting environmental conditions found in mosquito habitats during the rainy and dry seasons in a savannah area of Burkina Faso (West Africa).

**Results:**

Using scanning electron microscopy (SEM) and confocal imaging, we revealed significant changes in morphological features of the spiracles in females *An. gambiae s.l.* exposed to contrasted environmental conditions. Hence, the hairs surrounding the spiracles were thicker in the three species when raised under dry season environmental conditions. The thicker hairs were in some cases totally obstructing spiracular openings. Specific staining provided evidence against contamination by external microorganisms such as bacteria and fungi. However, only further analysis would unequivocally rule out the hypothesis of experimental artifact.

**Conclusion:**

Morphological changes in spiracular features probably help to limit body water loss during desiccating conditions, therefore contributing to insect survival. Differences between species within the *An. gambiae* complex might therefore reflect different survival strategies used by these species to overcome the detrimental dry season conditions in the wild.

**Electronic supplementary material:**

The online version of this article (doi:10.1186/s13071-015-1289-0) contains supplementary material, which is available to authorized users.

## Background

Habitats of tropical savannahs are characterized by pronounced seasonal and daily fluctuations in environmental conditions such as temperature (with hot days and cool nights) and relative humidity. The conditions for malaria transmission in these regions are suitable only during certain periods of the year, particularly in the rainy season [1]. Thus, the vector dynamics, reproductive period and disease transmission intensity fluctuate considerably with this seasonal variation in temperature, precipitation and day length [2–4]. During the unfavourable (dry) weather, malaria mosquitoes of the *Anopheles gambiae s.l.* species complex are exposed to severe desiccation challenge, either through the drying-up of their breeding sites and/or the low ambient relative humidity [5–7]. Additionally, in early dry season conditions, mosquito larvae might further experience increased crowding while available surface water collections shrink and vanish. To survive through unfavourable conditions, many insects undergo dormancy (diapause or quiescence), characterized by a suite of morphological, physiological, biochemical and behavioural changes that enhance tolerance and extend survival to environmental stresses [8, 9] and in particular to desiccation [10].

The two molecular forms of *An. gambiae s.s.,* recently named *An. coluzzii* (former M molecular form) and *An. gambiae* (former S molecular form) [11] and *An. arabiensis* are members of the *Anopheles gambiae s.l.* complex, a group of closely related and morphologically indistinguishable species [12]. Their distribution ranges encompass broad environmental and ecological settings, including arid and semi-arid areas. Although widely sympatric, the three species exhibit molecular, behavioural, physiological, and ecological differences [13–18]. Despite increased attention drawn in recent years on dry season survival strategies in these major African malaria vectors species [7, 19–21], little is known about the processes that sustains survival during the stressful dry season conditions. A recent field study showed evidence that aestivation (summer diapause) is one mechanism that allows *An. coluzzii* to persist in the Sahel [7]. However, migration to/from more favourable localities where reproduction continues year-round might also be involved [19].

Suppression of water loss is a characteristic of species that face weather-induced desiccation [22]. It is also known that the main route of water loss is the cuticle, while water loss during respiration accounts for about 5–20 % of the total water evaporation [23, 24]. Respiratory gas exchanges in mosquitoes occur through a multi-branched tracheal system, where cuticular openings called “spiracles” are located on the thorax and the abdomen. Spiracles are very variable structurally between genus and species, however, typically, the opening leads to a cavity (the atrium) from which the tracheae arise. In addition, spiracles of most insects have closing valves and can be surrounded by dust-catching hairs. In adult mosquitoes, spiracles are paired, bilaterally symmetric and located on the mesothorax, metathorax, and abdominal segments. Their apertures ensure the trade-off between gas exchanges and water loss [7], since oxygen, a necessary gas for cell activity, must pass through the spiracles to enter the respiratory system [25]. Regulation of spiracle aperture plays a role in water conservation and may best be illustrated in insects showing discontinuous respiration [26, 27]. Although the adaptive significance of discontinuous gas exchange (DGC) is a subject of considerable debate, this respiratory regimen is characterized by a period in which the spiracles are fully closed. Indeed, DGC is a repeating cycle of spiracular openings and closings that leads to periodic releases of carbon dioxide [28]. In ants *Pogonomyrmex barbatus,* the metabolic rates were found lowest for individuals using DGC, intermediate for individuals using cyclic gas exchange, and highest for individuals using continuous gas exchange [29]. Permanently opened spiracles allow maximum gas exchange but insects face desiccation stress more quickly [30]. Studying the effects of a xeric environment on water balance in *Glossina sp.*, Bushel [31] concluded that increased water retention in *Glossina* sp from xeric environments resulted largely from spiracular control of transpiration. Spiracle size could also be positively correlated with water loss. Indeed, Nagpal and collaborators [32] showed that the spiracles of ecological variants of *An. stephensi* displayed different sizes, being the smallest in the xerophilic ecotype.

Water conservation mechanisms may be of considerable importance to survival and there is evidence that spiracles are instrumental in water conservation while still responding to the often-conflicting demands of respiration. The object of our study was therefore to survey, using electronic and confocal microscopy, the effect of contrasted environmental conditions on spiracles morphology of adult females of the *An. gambiae s.l.* complex raised under environmental conditions mimicking those found in a savannah area of Burkina Faso during the rainy and the dry seasons.

## Methods

### Mosquitoes

We used mosquito colonies maintained at the Institut de Recherche en Sciences de la Santé (IRSS) insectaries in Bobo-Dioulasso under controlled conditions (27 ± 1 °C, 80 ± 10 % relative humidity (RH) and 12:12 dark/light cycle). The *An. arabiensis* colony originated from wild gravid females collected in Bobo-Dioulasso (11°10’ N, 4°17’ W) in 2008, the *An. coluzzii* colony was seeded from females collected in the village of Bama, Vallée du Kou (11°23’ N, 4°24’ W) in 2008, and the *An. gambiae* colony originated from females collected in the village of Soumousso (11°04’ N, 4°03’ W) in 2009 (see [18] for further details). All these sites are located in southwestern Burkina Faso, within 50 km from each other and were previously described [15, 18, 33].

### Environmental conditions, mosquito rearing and sample collection

Mosquitoes were reared from the egg to the adult stage in programmable climatic chambers (Sanyo MLR 315H, Japan), where the climatic parameters characterizing the rainy and the dry season conditions were defined from hourly averaged records collected in Bama during August 2010 (rainy season, RS) (Additional file 1: Figure S1A) and December 2010 (dry season, DS) (Additional file 1: Figure S1C) using a Vantage Pro2 monitoring station (Weatherlink; Davis Instruments, Hayward, CA, U.S.A.) As previously described [18, 34], twelve steps cycles were designed to reproduce as close as possible the natural climatic variations monitored in the fields. A photoperiod of 12 L: 12D was set, corresponding to day length at the end of the rainy season in Bama (i.e., mid/late October), when dry season survival strategies might be set up in sensitive stages. To control for potential cycle failure, temperature and relative humidity were recorded inside the chamber at a 10 min pace using MSR145 Data Loggers (MSR145B4HL, MSR electronics GmBH, Switzerland). We also recorded the temperature cycles to which larvae were exposed using waterproof MSR145B4T2L monitors. The daily air temperature and humidity fluctuations recorded in the climatic chamber, identical to [18, 34], are represented in Additional file 1: Figure S1B and D and water temperature variation is shown in Additional file 2: Figure S2.

For each mosquito colony, eggs were collected from three independent batches, each produced by >50 caged females. Eggs were transferred into transparent plastic trays (21.5 × 16 × 9.5 cm) containing spring water and incubated in the climatic chambers. After hatching, first-instars were counted and distributed into new plastic trays containing 1 L of spring water at a density of 100 larvae per tray. Each species was reared in separate pans, and three trays were used per colony and per environmental condition. The first two larval instars were fed every other day with 0.30 mg of ground fish food (Tetramin®), whereas later instars were supplied with 0.75 mg of food. Water was renewed when necessary to avoid scum formation and fouling of the media. The water used for renewal was stored in advance in the climatic chambers to avoid any perturbation of the temperature cycles. Every day, the position of the trays was randomly alternated to avoid positional effects within the incubators. Before adult emergence, pupae were collected with pipettes and transferred into plastic cups covered with netting. Emerging adults were immediately removed with an aspirator. Males were discarded and only females were kept in plastic cups closed with nets and fed using a cotton ball soaked with 10 % glucose solution. Starting from the first day after their emergence, the dry season (DS) females (inside the cups) were placed into large plastic boxes filled with desiccant (Silica gel Chameleon©), where relative humidity reaches values as low as 15 %, as measured by a T/H recorder MSR145 (MSR Electronics, GmBH, Switzerland). This was performed to mimic the field conditions, where relative humidity drops below levels allowed in the climatic chambers during the hottest hours in December (Additional file 1: Figure S1A). Hence, females were placed into the boxes (without access to sugar) at 10 a.m. and removed at 4 p.m., where a new cotton ball soaked with glucose solution was provided. For consistency, the same protocol was applied to the females raised under rainy season (RS) conditions, except that the boxes were filled with desiccant soaked with water. Environmental conditions monitored inside the boxes are provided in Additional file 1: Figure S1C and D. This was reproduced each day until the 10th day post emergence, where females of each species were collected around 2 p.m. at the very same time for each environmental condition to avoid potential confounding effect of circadian rhythms on spiracle aperture. Samples were snap frozen and preserved in 70 % ethanol under ambient temperature. Twenty alcohol-preserved specimens were sent to the University of Rennes 1, France, before being prepared for further processing through scanning electron microscopy (below).

A second experiment involving *An. coluzzii* and *An. gambiae* raised under the same dry season conditions as described above was further performed for confocal imaging (see below). This experiment involved 50 ethanol preserved mosquitoes from each species, which were sent to the University of Pierre et Marie Curie, Paris, France for spiracle staining and observations using confocal microscopy.

### Scanning Electron Microscopy (SEM)

Eight females from the first experiment were randomly picked for each species and each environmental condition and processed for SEM observations. The females were dehydrated by immersion during 30 min in ethanol/water solutions of graded ethanol concentrations (70, 80, 90, 95 and 100 %), according to [35 ]. Mosquitoes were then incubated in acetone and kept until use. They were dried by the critical point method using liquid CO_2_ in a Balzers Critical Point Drier (Balzers Union FL-9496 Balzers/Furstentum Liechtenstein, Germany) apparatus attached to specimen holders and coated with gold and palladium in a sputter coater (FINE COAT ion sputter JFC-1100, JEOL, Japon). Finally, the specimens were observed under a scanning electron microscope (JEOL SJS-6301F, Japan).

### Confocal imaging and spiracle staining

Females of the second experiment and those from the field (for which the species status was assessed using PCR technique [36]) were dedicated to the confocal imaging only, for which mosquito thoraces were dissected in Phosphate Buffer Saline (PBS) and fixed for 30 min in PBT (PBS + 0.1 % Triton X-100) containing 4 % of paraformaldehyde.

The thoraces of 25 randomly picked females from experiment 2 (seven from the species *An. coluzzii*, 18 from *An. gambiae*, see Table 1) were further stained with a mixture containing Phalloidin (a commonly used stain for F-actin filaments) and TO-PRO®-3 (a commonly used stain for nucleic acids): after three washes of 10 min in PBT, thoraces were stained overnight at 4 °C in PBT containing Alexa Fluor® 568 Phalloidin (0.07 μM) and TO-PRO®-3 (0.01 mM). Samples were rinsed 3 times for 10 min in PBT then hemi-thoraces were mounted with spiracles facing the coverslip in DABCO (Sigma) and examined by confocal microscopy using a Nikon (TE 2000-U) microscope.

**Table 1:** Number of opened and closed spiracles and number of spiracles displaying coated setae when observed under confocal microscope after staining with the vital stain TO-PRO-3. Unobservable spiracles are those for which the dissection and/or mounting steps before observation were unsuccessful. Observed mosquitoes were from experiment 2

## Results

### Morphological variation between species and environmental conditions

Entire females from experiment 1 were carefully observed under the SEM in order to look for striking morphological and structural differences between species and/or environmental conditions, with a focus on the respiratory system. The only differences that jumped out during our observations resided in the structural appearance of the spiracle apparatus.

**Fig. 1:**
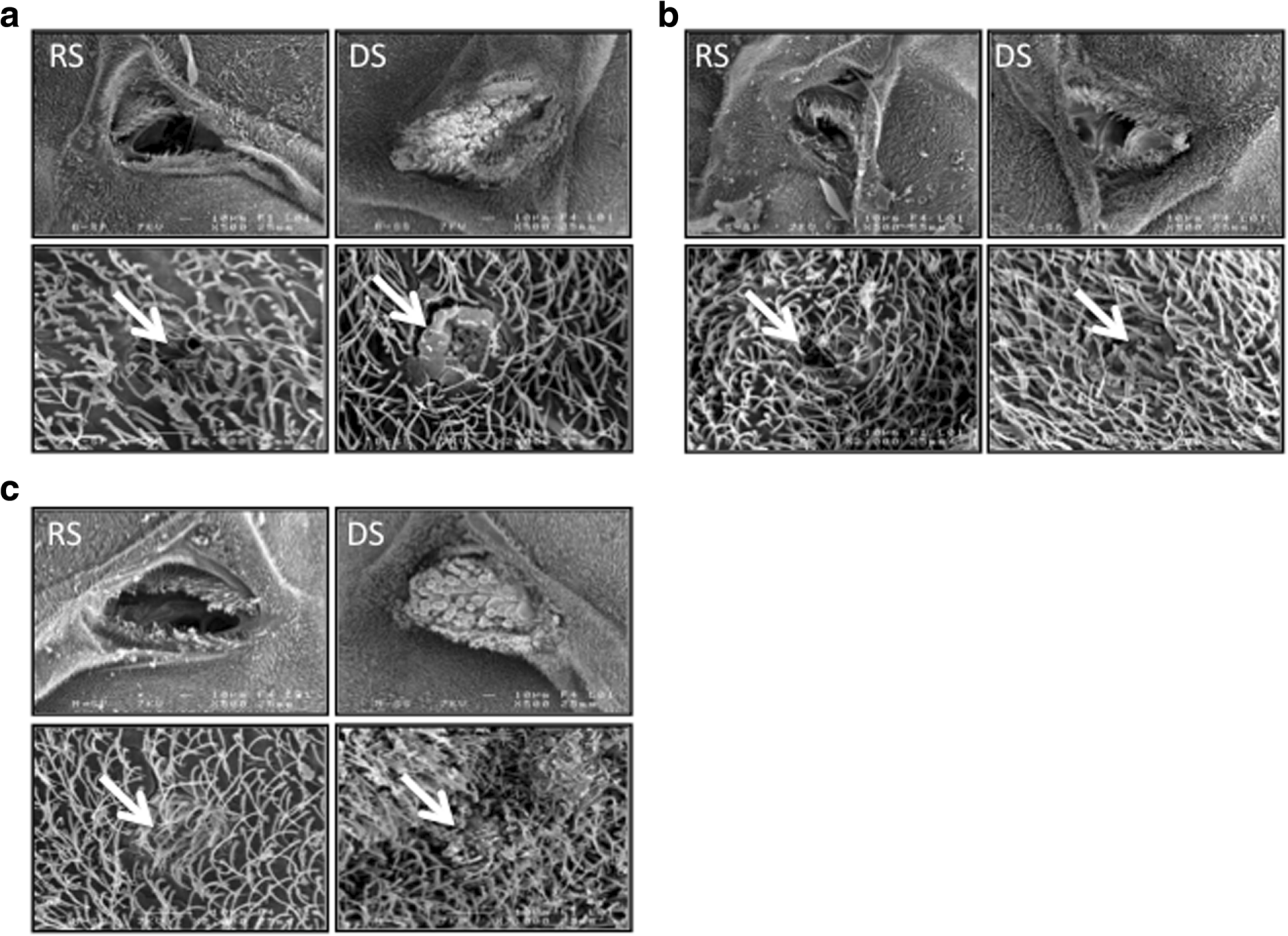
Observation using SEM imaging of mesothoracic and abdominal spiracles of *An. gambiae s.l.* females raised under different environmental conditions (DS: Dry Season; RS: Rainy Season). Spiracle features surrounding spiracles are thickened under dry season conditions. White arrows point to the thickening of the spiracular features. **a**.: *An. arabiensis*; (**b**).: *An. gambiae*; (**c**).: *An. coluzzii*

**Fig. 2:**
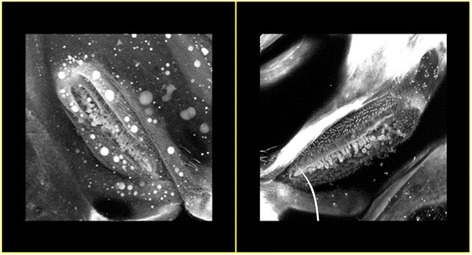
Observation using confocal imaging showing a meta-thoracic spiracle with thickened hairs in two females *An. coluzzii* raised under dry season conditions

The three species displayed differences in the visual aspect of the trichomes (or setae) of the mesothoracic spiracles in females reared in DS conditions compared to those reared in RS conditions (Fig. 1). Indeed, our observations showed that in 100 % of the females observed under the SEM, the mesothoracic spiracles are wide open in females reared in RS conditions whereas the hairs appear wider and thicker in females reared under DS conditions, ultimately plugging entirely the spiracular aperture in *An. coluzzii* and *An. arabiensis* (Figs. 1 and 2). The phenotype is less striking in *An. gambiae* females, where the hairs, although oversized, leave a wide aperture. We were able to observe abdominal spiracles in only 4–5 females per species and conditions; however, all the spiracle structures observed in DS females were modified, and displayed obstructed apertures (Fig. 1). This suggests that the morphological modification of spiracular associated structures applies to the whole respiratory system. We confirmed our results in a second experiment where meso- and metathoracic spiracles of 25 females raised under DS conditions were observed under confocal imaging (Fig. 2, Table 1). Overall, about 63 % of *An. gambiae* (*N* = 18) and 28 % *An. coluzzii* (*N* = 7) females displayed oversized hairs around their thoracic spiracles under dry season conditions (Table 1). Thickened hairs were not always associated with the mechanical closure of the spiracular valves and both phenotypes can be independent although a significant trend appears. Of all the spiracle structures showing oversized hairs, 57 % (17 out of 30) were mechanically closed whereas this percentage was 28 % (25 out 90) for the spiracles without coating (χ^2^_1_ = 7.033, *p* = 0.008).

### Spiracle staining, observations, and related hypothesis on the origin of the oversized hairs

**Fig. 3:**
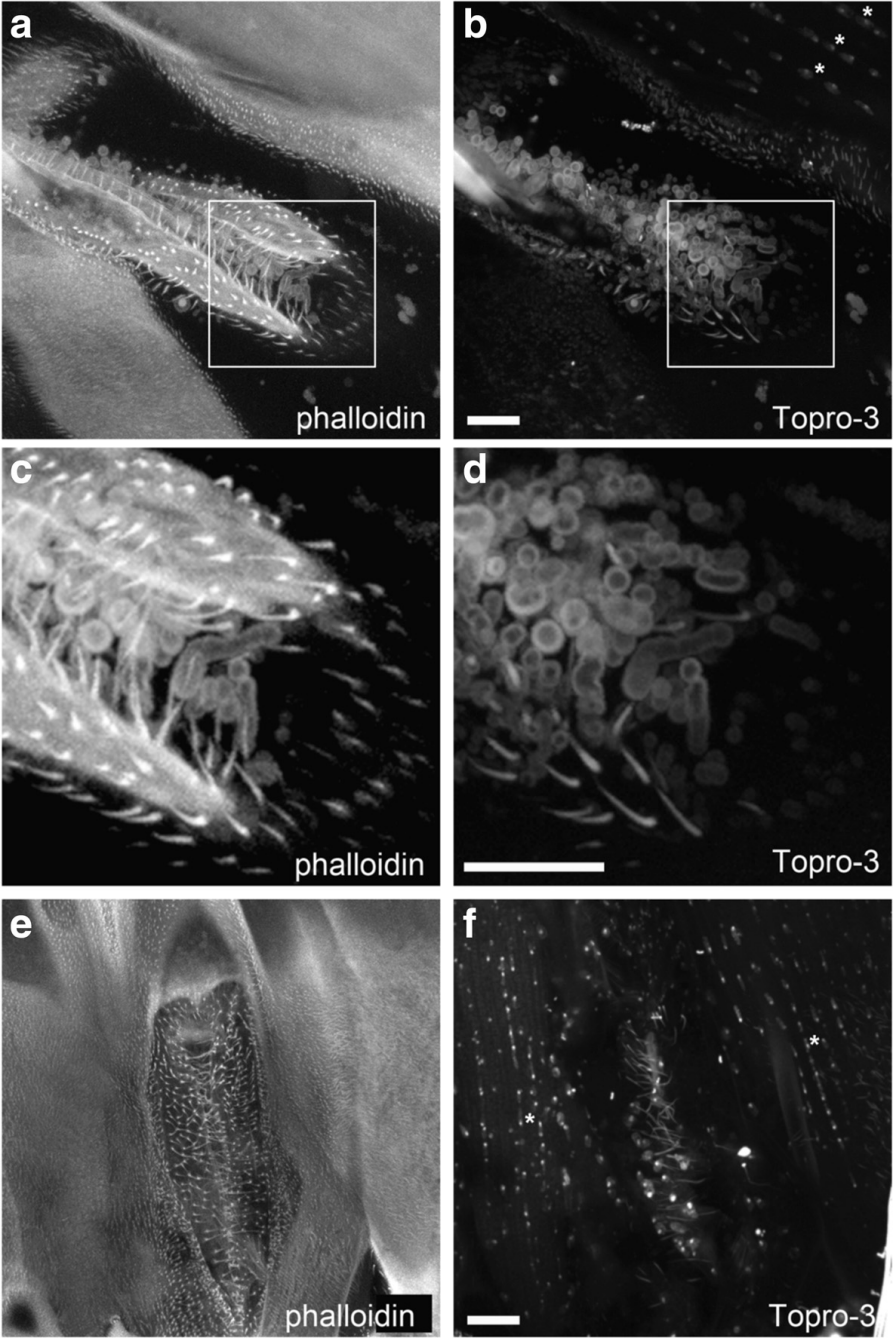
Phalloidin (**a, c **and **e**) and TO-PRO-3 (**b, d **and **f**) staining of spiracles and thick setae in *An. coluzzii* females raised under dry season conditions. Spiracle structures were observed using confocal imaging

In all the mosquitoes we observed, neither Phalloidin (which stains F-actin filaments) nor TO-PRO®-3 (which stains nucleic acids) probes gave specific signals around or in the thickened hairs, whereas for TO-PRO®-3, a specific staining was detected in the nuclei of mosquitoes’ muscles (Fig. 3, asterisks). This staining is taken as our positive control and rules out a potential failure in the staining or detection procedures to explain the lack of signal in the setae.

Over 50 photographs under confocal imaging were taken that allowed the following observations: (i) in spiracles for which setae are not oversized, we can see that these structures are rooted in the insect’s cuticle (Fig. 3e–f), (ii) setae are present at similar densities in all spiracles structures, whether they are oversized or not (Fig. 3f).

The lack of live cell specific staining, the observation that the oversized setae are differentiated from the mosquito cuticle and the fact that setae’s densities between and specimen are equivalent among all the observed specimens, are all evidence against the hypothesis of this structure being built by the aggregation of contaminating microorganisms like bacteria or fungi. However, only further analysis of the qualitative nature of these structures would unequivocally rule out the hypothesis of experimental artifact.

## Discussion

Despite the epidemiological importance of *Anopheles* mosquitoes, little is known about the mechanisms underpinning the survival of these species during the harsh conditions of the dry season in Africa. Here, we provide evidence for morphological variation in species of the *An. gambiae s.l.* complex in response to environmental variations*.* Because desiccation threat is more severe when spiracles are open [37, 38], we expected that mosquitoes reared in low ambient humidity and high temperatures, as typically observed during the dry season in sub-Saharan Africa, would exhibit morphological traits that serve as adaptations to reduce water loss. In fact, water is lost rapidly through opened spiracles during respiration [12, 39, 40]. In the arid-adapted ant *Cataglyphis bicolor*, the thoracic spiracles act as high-capacity gateways to the tracheal system and are responsible for approximately 90 % of the total gas exchange including water vapour during running activity [41]. Moreover, studies from other insects have demonstrated that spiracle closure greatly facilitates water conservation [37, 42–45]. For example, by measuring the rate of water loss of tsetse flies in varying states of desiccation, Bursell [37] was able to show that spiracular control of transpiration increased as water reserves decreased.

The ability to close spiracles mechanically using the valve mechanism is a physiological adaptation that reduces water loss in insects [38, 46–48]. Hence, closed spiracles have been reported in *Drosophila melanogaster* during flight to reduce water loss and gas exchange into the tracheal system [38]. Although functional experiments are lacking to link the size or shape of the thoracic or abdominal spiracles with heat and/or desiccation tolerance, it is nonetheless clear that spiracular structures evolve towards smaller sizes in mosquito species inhabiting dry climates [49]. For instance, in the hygrophilous species of *Anopheles* and *Aedes*, spiracles are generally larger, whereas in the xerophilous species the openings are much smaller [30, 49]*.* Moreover, the spiracular index*,* i.e. the ratio of the length of the spiracle to the length of thorax has been used as a tool to identify ecological variants exposed to contrasted environments in *An. stephensi*, this ratio being smaller in xeric *vs* mesic environments [32].

The mechanical closure of spiracles in desiccating conditions represents a rapid and transient response, which is thought to be regulated by a sensory mechanism elicited by low relative humidity [32]. In contrast, the specific spiracular structure modifications we observed through the whole respiratory system might represent important long-term adaptation, which could be programmed in anticipation to DS conditions. Hence, the ability to build oversized hairs that ultimately plug the spiracle apertures could be triggered by environmental cues sensed during the aquatic stages (i.e. high temperatures) or right upon emergence of adult mosquitoes (i.e. high temperatures and/or low relative humidity). Trichomes of various size, shape and density have been reported to line the spiracles atrium cavity in Geophilomorpha species, these structures being described as “solid and expended distally and showing a network of sclerotisation” in *Cormocephalus calcaratus*, “flap-like” in *Strigamia*, “cone shaped” in *Geophilus insculptus*, or “elongated plates” in *Hapiophilus subterraneous* (Lewis, [50] and references therein). In his attempts to provide a functional explanation to such variability, Lewis [22], reported a strong correlation between the presence of a thick layer of trichomes in the atrium and resistance to desiccation challenge in 4 geophilomorphs. He concluded that this structure, together with a narrowed spiracular opening, might limit water evaporation from the atrium. Outgrowths cover the spiracular openings of the xerophilous buprestid beetle and, in the cockroach, such structures line the atrium, either outside or inside the valves (Hadley, [23] and references therein). Oversized setae around the spiracles may therefore well play the same role in mosquitoes than in the above-cited species, and might contribute to maintaining minimal metabolic activity while minimizing respiratory water loss. As such, they could be part of the survival strategies developed by *An. gambiae* s.l. to cope with low relative humidity values encountered during the dry season, either under diapause, quiescence or reproductively active states.

However, to our knowledge, our study is the first to report trichomes variability within the same species, putatively induced by environmental triggers. Dry season specific phenotype of the spiracles’ setae was also observed in preliminary experiments involving mosquitoes collected in Bama at the beginning of the dry season. Two out of 22 mosquitoes observed under confocal imaging were showing oversized setae (i.e. 9 %), however, the phenotype was less striking in the sense that very few setae were indeed oversized (data not shown), which might suggest a progressive building of the phenotype over time, either with mosquito age and/or with the installation of the dry season. These preliminary observations warrant further investigations under field and/or semi-field conditions that will help resolve the biological meaning of such phenotypes as well as its underlying physiological and ecological determinants. Nonetheless, given that such structural variation has not been observed in any *Anopheles* mosquito to date, further experiments must also be conducted to strengthen our findings and to definitely rule out the caveat of an experimental artifact. Among these, a time course observation of the progressive building of oversized trichomes, careful observation of a recent study on *Drosophila melanogaster* revealed that lipid deposits are used to waterproof the spiracles [51], a link that might also exist in *An. gambiae s.l.* mosquitoes.

The ability to manage water reserves through the modification of spiracular morphology might account, at least in part, for better survival under desiccation challenge and increased body water content observed in mosquitoes when raised under dry season conditions [52]. In addition to water conservation, reduced spiracle openings might also contribute to lower metabolic rate and gas exchange through respiration. Huestis and collaborators [20] indicated that the mean metabolic rate of *An. coluzzii* was lowest during the transition period between the wet and the dry season in the Sahel, which is consistent with obstructed spiracles as we observed in mosquitoes reared under DS conditions.

## Conclusion

This study identified morphological variations in *An. gambiae s.l.* mosquitoes when exposed to the severe dry season conditions in West African savannahs. These morphological changes might reflect specific adaptations to increase survival under different climatic or micro-climatic conditions, pointing towards an important influence of spiracles’s hairs on the rate of respiratory water loss and slowing down of the global metabolism. Although there is clear evidence for seasonal differentiation in *An. gambiae s.l.* species, further research including cuticle morphology and composition and gas exchange rates are required to explore in more detail the biological relevance and adaptive value of these morphological adaptations.

## Additional files

Additional file 1: Figure S1. Daily environmental conditions of temperature (dashed line), relative humidity (solid line), and dark/light duration (horizontal bars) recorded during the wet season in the field (A) inside the climatic chambers (B) and at the onset of the dry season in the field (C) and inside the climatic chambers (D). The red arrows indicate the times when females inside the cups were placed into large plastic boxes filled with desiccant (10 h) and were removed (18 h). Between the arrows, temperature and relative humidity values are those recorded inside the plastic boxes. Humidity values inside the climatic chamber are 2–5 % above field ones for the rainy season conditions due to condensation problems in climatic chambers at higher humidity values. (TIF 870 kb) Additional file 2: Figure S2. Daily temperature of the larval rearing water inside climatic chambers; wet season conditions (dashed line) and dry season conditions (solid line). (TIF 547 kb)

## Abbreviations

DS: Dry Season
RS: Rainy Season
IRSS: Institut de Recherche en Sciences de la Santé
WHO: World Health Organisation
RH: Relative Humidity
SEM: Scanning Electron Microscopy
