# Supplementary figures and images for: Morphological changes in the spiracles of Anopheles gambiae s.l (Diptera) as a response to the dry season conditions in Burkina Faso (West Africa)
Source: Parasit Vectors. 2016 Jan 7;9:11. doi: 10.1186/s13071-015-1289-0 (PMC4704408; doi:10.1186/s13071-015-1289-0)

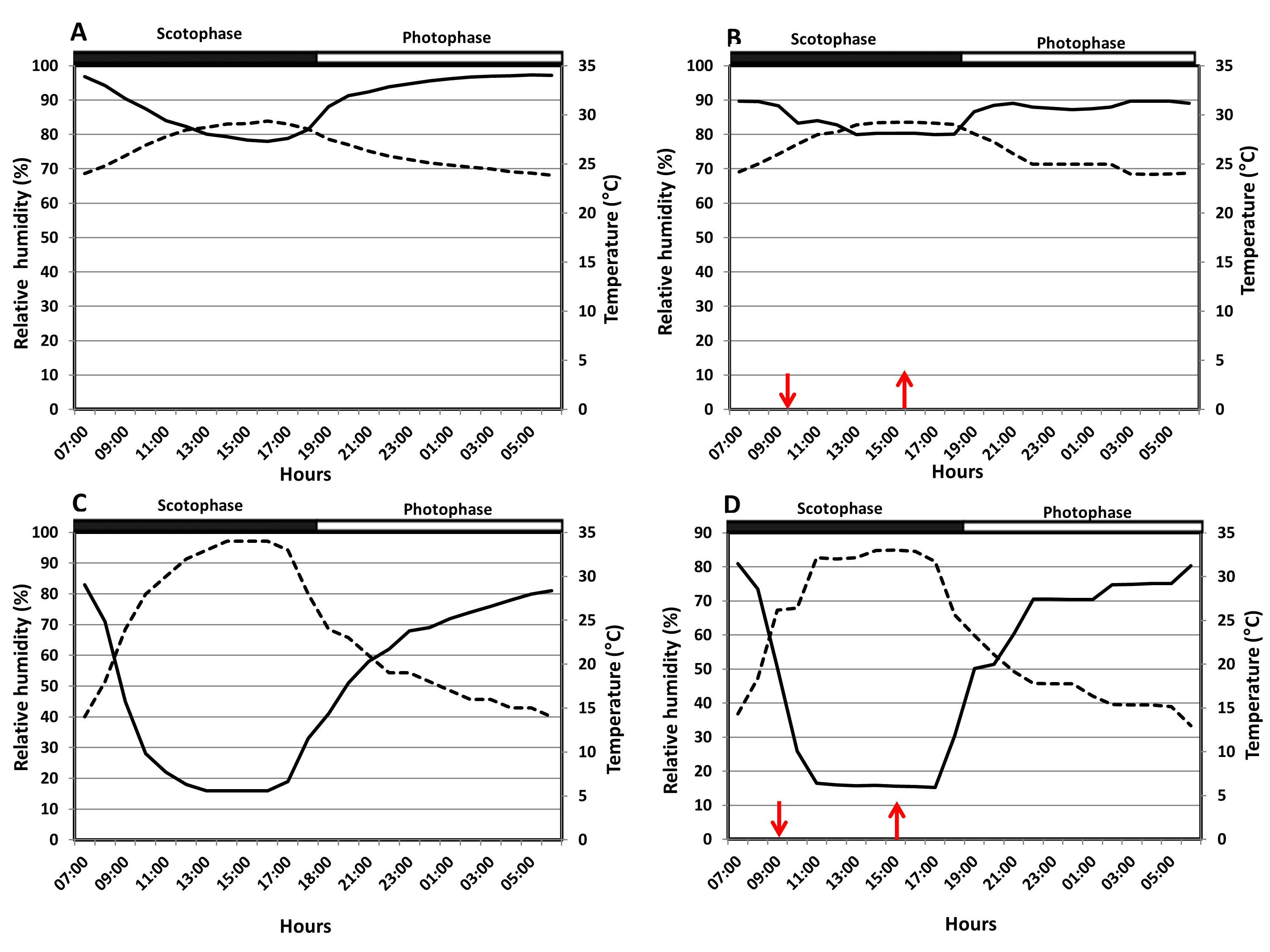

Supplement: Additional file 1: Figure S1. — Daily environmental conditions of temperature (dashed line), relative humidity (solid line), and dark/light duration (horizontal bars) recorded during the wet season in the field (A) inside the climatic chambers (B) and at the onset of the dry season in the field (C) and inside the climatic chambers (D). The red arrows indicate the times when females inside the cups were placed into large plastic boxes filled with desiccant (10 h) and were removed (18 h). Between the arrows, temperature and relative humidity values are those recorded inside the plastic boxes. Humidity values inside the climatic chamber are 2–5 % above field ones for the rainy season conditions due to condensation problems in climatic chambers at higher humidity values. (TIF 870 kb) [file 13071_2015_1289_MOESM1_ESM.tif]

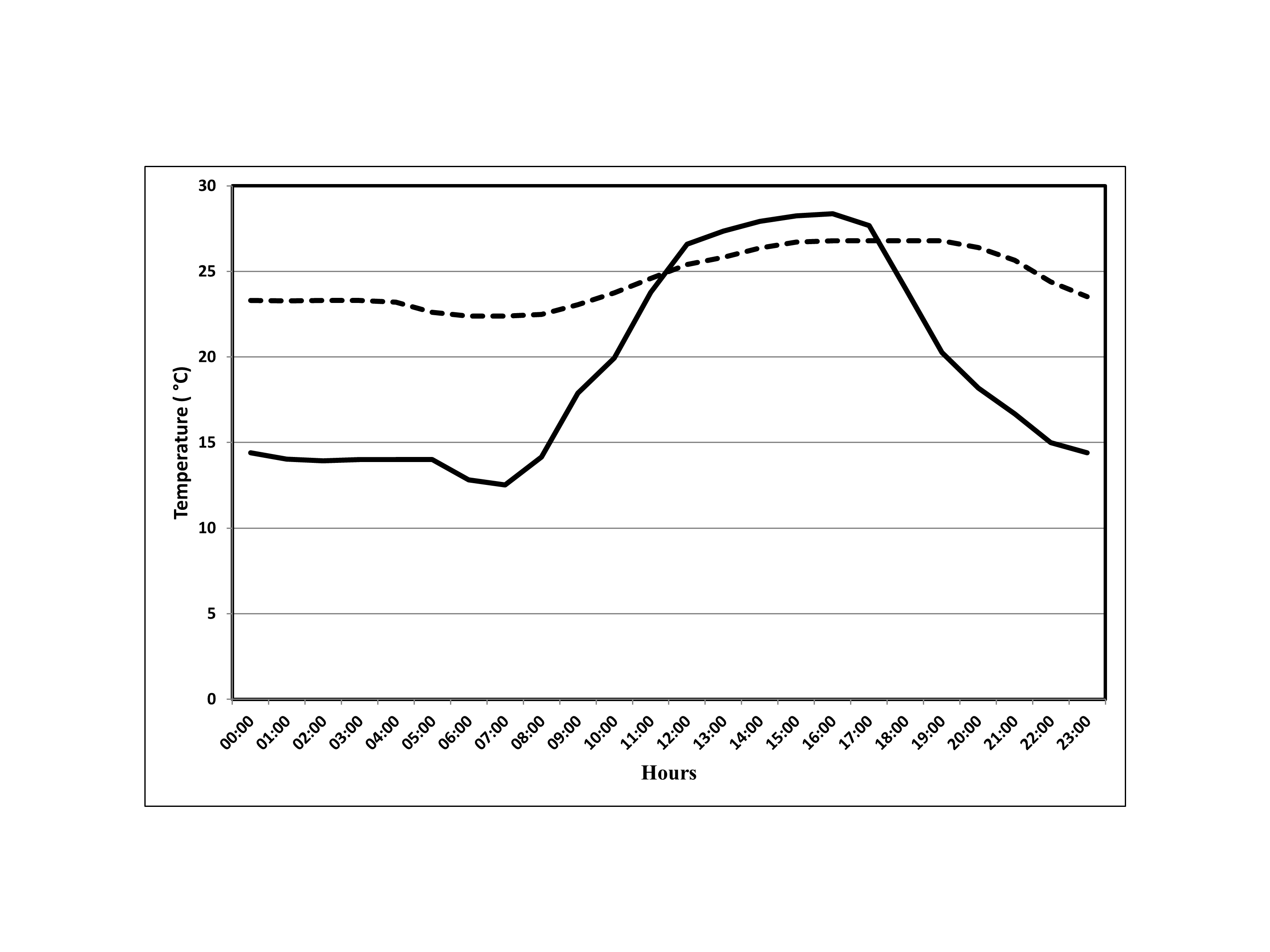

Supplement: Additional file 2: Figure S2. — Daily temperature of the larval rearing water inside climatic chambers; wet season conditions (dashed line) and dry season conditions (solid line). (TIF 547 kb) [file 13071_2015_1289_MOESM2_ESM.tif]
